# Prediction of 28-day mortality by indocyanine green disappearance rate, other markers of hepatic function and transpulmonary thermodilution parameters: a prospective study in 154 patients

**DOI:** 10.1186/cc12117

**Published:** 2013-03-19

**Authors:** W Huber, D Ertekin, T Langer, B Saugel, T Lahmer, M Messer, C Spinner, C Schultheiss, RM Schmid

**Affiliations:** 1 Klinikum rechts der Isar, Technical University of Munich, Germany

## Introduction

Hepatic dysfunction has been associated with outcome of ICU patients. However, most scoring systems including APACHE II only marginally reflect acute liver dysfunction on admission. Indocyanine green (ICG) is eliminated by hepatobiliary excretion. Therefore, the ICG plasma disappearance rate (ICG-PDR) is used as a dynamic liver function test. ICG-PDR has been associated with mortality in several studies.

## Methods

A prospective study to compare prediction of 28-day mortality by ICG-PDR, other markers of liver function and scoring systems (primary endpoint). In the subgroup of patients with transpulmonary thermodilution (TPTD) monitoring (PiCCO device; Pulsion, Munich, Germany), predictive capabilities of ICG-PDR were compared with cardiac index (CI), extravascular lung water index (EVLWI), global end-diastolic volume index (GEDVI) and pulmonary vascular permeability index (PVPI). ICG-PDR (i.v. bolus of 0.25 mg/kg ICG; LiMON device, Pulsion) and all other predictors were determined within 48 hours after admission. Statistics: IBM SPSS 20.

## Results

A total of 154 patients (46 female, 108 male), age 59 ± 13 years, APACHE II score 16.0 ± 5.7, SOFA score 7.6 ± 4.2. Etiology: sepsis 14.4%, cirrhosis 28.8%, GI bleeding 8.9%, ARDS 17.8%, cardiogenic shock 4.1%, acute renal failure 3.4%, various 22.6%. The 28-day mortality was significantly predicted by APACHE II (ROC-AUC: 0.762; *P <*0.001) and SOFA (AUC: 0.784; *P <*0.001). Among markers of hepatic function on admission, ICG-PDR provided the largest AUC (0.742; *P <*0.001), which was larger than for GOT (AUC: 0.646; *P *= 0.019), bilirubin (AUC: 0.641; *P *= 0.023) and INR (AUC: 0.614; *P *= 0.067). Among TPTD parameters, only PVPI significantly predicted 28-day mortality (AUC: 0.643; *P *= 0.043), whereas CI, GEDVI and EVLWI were not predictive. Prediction of 28-day mortality by SOFA could not be improved by models including any hepatic parameter. By contrast, ICG-PDR was independently (*P *= 0.036) associated with mortality when included in a model (*R *= 0.58) with APACHE II. This model based on APACHE II and ICG-PDR provided the largest of all ROC-AUCs (AUC: 0.804; *P <*0.001). ICG-PDR itself was independently associated with age (*P *= 0.025), but not with any other biometric parameter (gender, weight and height).

## Conclusion

ICG-PDR on admission is an independent predictor of 28-day mortality. Predictive capabilities particularly of APACHE II can be improved by combination with ICG-PDR. Among TPTD-derived parameters, only PVPI provides significant prediction of mortality.

